# The Y plane is a reliable CT-based reference for glenoid component positioning in shoulder arthroplasty

**DOI:** 10.1186/s40634-022-00481-z

**Published:** 2022-05-18

**Authors:** Jean-Marc Glasson, Floris van Rooij, Luca Nover, Mo Saffarini, Jean Kany

**Affiliations:** 1 Clinique Saint François, ELSAN, 22 Avenue Marcel Lemoine, 36000 Châteauroux, France; 2 Clinique du Parc Imperial, 28 Boulevard du Tzarewitch, 06000 Nice, France; 3 ReSurg SA, Rue Saint Jean 22, 1260 Nyon, Switzerland; 4 Clinique de l’Union, Ramsay Santé, Boulevard de Ratalens, 31240 Saint-Jean, France

**Keywords:** Y-point, Computed tomography, RSA, TSA, Lateralization

## Abstract

**Purpose:**

To determine the reliability of anatomic references for mediolateral component positioning in shoulder arthroplasty.

**Materials and methods:**

The computed tomography scans of 86 shoulders free of arthritic or anatomic deformities were studied. Two surgeons independently digitized a series of points, including the intersection of the 3 bone branches of the scapular spine (Y), the center of the glenoid surface (G), the most medial point of the scapula (MS), the cortical convergence (CC) of the anterior and posterior margins of the glenoid, the base of the coracoid (BC), the anterior (HA) and posterior (HP) margins of the subchondral bone.

**Results:**

The mean mediolateral distances between G and Y, BC, CC were respectively − 19.6 mm, − 1.5 mm, and − 36.8 mm. The consistency of anatomic landmarks was greatest for Y (standard deviation (SD) =2.3 mm; interquartile range (IQR) =3 mm), compared to BC (SD = 4.6 mm; IQR = 7 mm), and CC (SD = 6.6 mm; IQR = 8 mm). The repeatability of anatomic landmarks was excellent for all measurements. The mean ratios (relative to humeral head size) of distances between G and Y, BC, CC were respectively − 0.45, − 0.04, and − 0.85. The consistency of ratios was greatest for Y (SD = 0.05; IQR = 0.06), compared to BC (SD = 0.11; IQR = 0.14), and CC (SD = 0.13; IQR = 0.17). The repeatability of ratios was excellent for Y and BC, while it was good for CC.

**Conclusions:**

The Y-plane is a reliable reference for glenoid component positioning in shoulder arthroplasty, with a consistent distance from the center of the glenoid surface, and could therefore be suitable for preoperative planning.

**Study design:**

Level III, comparative anatomic study.

## Background

Adequate lateralization of the humerus is important to grant sufficient stability [4, 5, 15], increase range of motion [14, 17, 20] and maximize survival of shoulder arthroplasty [16]. Several anatomic landmarks and axes have been established for preoperative planning and intraoperative adjustment of glenoid baseplate positioning [2, 11], but only one study [13] so far suggested a landmark specifically for lateralization; namely the base of the coracoid (BC). While the authors found BC to be sufficiently reliable, it may not always be tangible and discernible in cases with severe glenoid bone loss, or during revision surgeries.

The Y-plane, corresponding to the most lateral parasagittal image on which the scapular spine is in contact with the scapular body, has already been described for the assessment of rotator cuff muscle integrity and fatty infiltration [8, 18]. The Y-plane is considerably more medial than the BC, and therefore could be less affected by severe glenoid bone loss. In the authors’ experience, the Y-plane is also easy to locate, and represents a consistent landmark for implant positioning, though there are no published studies regarding its consistency and repeatability as a reference for shoulder arthroplasty.

The purpose of this study was to determine the reliability of the Y-plane as a reference for mediolateral component positioning in shoulder arthroplasty compared to other landmarks [2, 11, 13]. The hypothesis was that, in normal healthy shoulders, the mediolateral distance between the Y-plane and the center of the glenoid surface would have the greatest consistency and repeatability. The ultimate goal would be to prescribe appropriate lateralization of the glenoid component to improve the accuracy and utility of preoperative 3D templating software and surgical navigation, both of which are increasingly accessible and used by shoulder surgeons.

## Methods

In this retrospective imaging study, 103 consecutive shoulders were selected from their database of pre-existing computed tomography (CT) scans, assessed between January and June 2019 for rotator cuff tears, instability, and acromioclavicular joint pathology. All patients provided informed consent to use of their images and data for research and publishing purposes, and the study was approved by the institutional review board (IRB) in advance (COS-RGDS-2021-05-007-KANY-J). All scans were acquired using a 64 slice CT scanner with slice thickness of 0.625 mm to 1.000 mm, and included axial images from the superior margin of the acromion until the inferior third of the scapula. Upon preliminary assessment, the authors excluded 8 shoulders that had eccentric arthritis or other bony deformities, and 9 shoulders that had distorted (e.g. poor resolution due to artefacts) or incomplete images (e.g. medial margin of the scapula not visible). For the remaining 86 shoulders the Digital Imaging and Communication in Medicine (DICOM) scans were imported into the image processing software OsiriX (Pixmeo, Bernex, Switzerland) in standard resolution. This imaging software enabled simultaneous visualization of CT cross-sections in the coronal, sagittal and axial planes. The software enables direct measurement of dimensions and angles in all three planes.

**Fig. 1:**
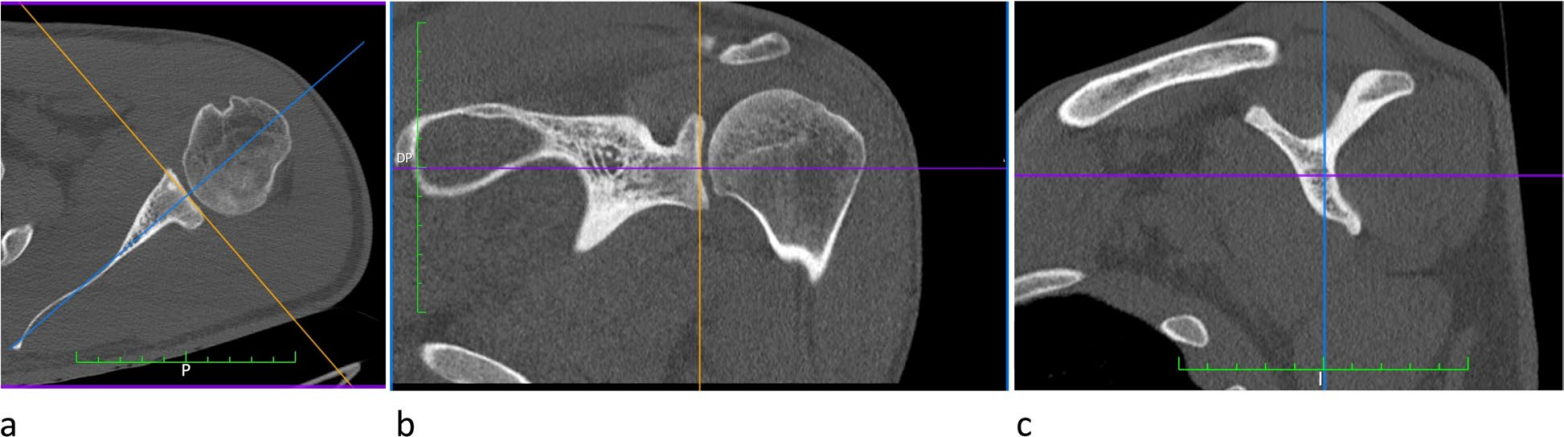
The reference planes were defined using the 3D multi-planar reformat (MPR) mode to enable direct measurement of dimensions and angles in all three planes: **a** An axial slice passing close to the glenoid equator was used to locate the scapular axis, passing through the medial border of the scapula and the centre of the glenoid; **b** The coronal view was used to locate the glenoid equator more accurately, and the corresponding axial slice was used to finetune the scapular axis; **c** The para-sagittal view (parallel to glenohumeral joint space) was used to locate the ‘Y-plane’, by scrolling from lateral to medial until both superior and inferior cortices of the scapular spine were in continuity with the body of the scapula

For each patient, the age, sex, and main pathology were noted, and two senior shoulder surgeons (JMG & JK) independently digitized a series of points, to enable assessment of inter-observer repeatability. The reference planes were defined using the 3D multi-planar reformat (MPR) mode:

1. An axial slice passing close to the glenoid equator was used to locate the scapular axis (SA), passing through the medial border of the scapula and the centre of the glenoid (close to the Friedman line) (Fig. 1a).
2. A coronal view passing through the SA was then used to locate the glenoid equator more accurately, and the corresponding axial slice was used to finetune the scapular axis [11] (Fig. 1b).
3. The para-sagittal view (perpendicular to the SA) was finally used to locate the ‘Y-plane’, by scrolling from lateral to medial until both superior and inferior cortices of the scapular spine were in continuity with the body of the scapula [8, 18] (Fig. 1c).

**Fig. 2:**
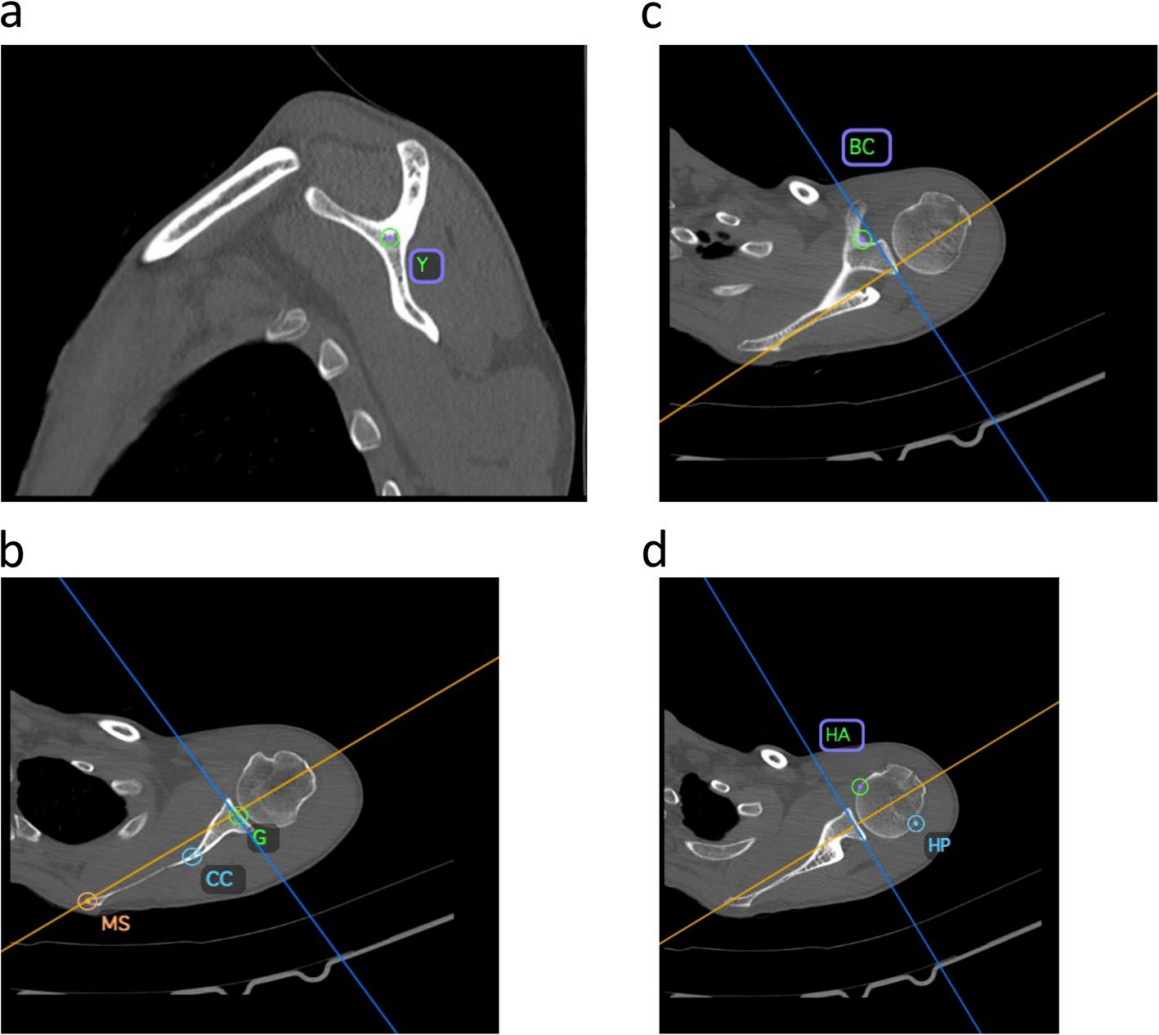
The 7 following points were digitized and named on each CT scan: **a** The Y point was digitized in the parasagittal view, at the intersection of the 3 bone branches (Y) on the aforementioned ‘Y-plane’; **b** The centre of the glenoid (G), the most medial point of the scapula (MS), and the cortical convergence (CC) of the anterior and posterior margins of the glenoid were all digitized in the same axial plane; **c** The base of the coracoid (BC) was digitized where the lateral cortex of the coracoid process merges with the cortex of the scapula in the axial view; **d** The anterior (HA) and posterior (HP) margins of the humeral head were digitized in the axial plane where the humeral head appeared to have the greatest size

The 7 following points were digitized and named on each CT scan:

- In the parasagittal view, the intersection of the 3 bone branches (Y) on the aforementioned ‘Y-plane’ (Fig. 2a).
- In a single axial plane, the centre of the glenoid surface (G), the most medial point of the scapula (MS) (both on the scapular axis), and the cortical convergence (CC) of the anterior and posterior margins of the glenoid (Fig. 2b).
- In the axial plane where the lateral cortex of the coracoid process merges with the cortex of the scapula, the base of the coracoid (BC) [13] (Fig. 2c).
- In the axial plane where the humeral head exhibits its largest size, the anterior (HA) and posterior (HP) margins of the subchondral bone (Fig. 2d).

For each CT scan, the cartesian coordinates of the digitized points were exported as comma separated variables (CSV) files, which were then imported into a spreadsheet using Excel (Microsoft, Redmond, Washington), and represented as an array for each shoulder. The origin was defined as the center of the glenoid surface (G), and a scapular axis was defined as the line connecting the center of the glenoid surface (G) to the most medial point of the scapula (MS). This was performed in 2 separate steps of mirroring (to represent all right shoulders as left shoulders) and rotation (to align point MS on the same horizontal axis as point G). During the steps of mirroring and rotation, the 3D coordinates of each point were available, though most visual representations only displayed the 2D coordinates (in the axial plane). The orientation of the scapula within the scanner was noted. The humeral head size was defined by the anterior (HA) and posterior (HP) margins of the subchondral bone on the axial slice where the humeral head appeared to have the greatest size. The mediolateral distances (parallel to/along the scapular axis) between points G and Y, CC, and BC were calculated in millimeters, and as ratios relative to the humeral head size. Positive values indicated that points were lateral to the glenoid, whereas negative values indicated that points were medial to the glenoid.

### Statistical analysis

Descriptive statistics were used to summarise the findings, and Shapiro–Wilk tests were used to assess the normality of data distributions. For normally distributed continuous data, differences between groups were evaluated using unpaired t-tests. For non-Gaussian continuous data, differences between groups were evaluated using Wilcoxon rank sum tests (Mann Whitney U test). Agreement between the 2 surgeons was calculated using intra-class correlation coefficients (ICC), which can be interpreted as follows: < 0.40, poor; 0.41–0.59, fair; 0.60–0.74, good; 0.75–1.00, excellent [6]. Statistical analyses were performed using R version 3.4.3 (R Foundation for Statistical Computing, Vienna, Austria).

## Results

The 86 shoulders (86 patients) studied comprised 49 right shoulders, and 37 left shoulders, from 50 men and 36 women. Their mean age was 49.3 ± 18.2 years (range, 15-84); two female adolescents aged 15 and 18 were included as they were deemed skeletally mature. The pathologies identified were rotator cuff tear limited to 2 tendons in 46 (53%), instability without significant humeral or glenoid bone loss in 26 (30%), mild centered glenohumeral arthritis in 9 (10%), and acromioclavicular joint dislocation in 5 (5.8%).

The mean humeral head size was 43.4 ± 4.0 mm (range, 36-52) (Table 1). The mean mediolateral distances between point G and points Y, BC, CC were respectively − 19.6 mm, − 1.5 mm, and − 36.8 mm. The consistency of anatomic landmarks was greatest for point Y (SD = 2.3 mm; IQR = 3 mm), compared to point BC (SD = 4.6 mm; IQR = 7 mm), and point CC (SD = 6.6 mm; IQR = 8 mm) (Fig. 2). The repeatability of anatomic landmarks was excellent for all measurements. The mean ratios (relative to humeral head size) of distances between point G and points Y, BC, CC were respectively − 0.45, − 0.04, and − 0.85. The consistency of ratios was greatest for point Y (SD = 0.05; IQR = 0.06), compared to point BC (SD = 0.11; IQR = 0.14), and point CC (SD = 0.13; IQR = 0.17). The repeatability of ratios was excellent for points Y and BC, while it was good for point CC.

**Table 1:** Radiographic measurements and interobserver agreement

					Inter-observer agreement	
Variable	Mean ±SD	Median	(range)	(IQR)	ICC	95% C.I.
**Measurements**						
Humeral head size *(mm)*	43.4 ± 4.0	43.6	(36 – 52)	(40 – 47)	0.89	(0.85 – 0.92)
G – Y	-19.6 ± 2.3	19.5	(-26 – -15)	(-21 – -18)	0.82	(0.74 – 0.88)
G – BC	-1.5 ± 4.6	-2.0	(-13 – 9)	(-5 – 2)	0.86	(0.81 – 0.90)
G – CC	-36.8 ± 6.6	-35.7	(-60 – -23)	(-40 – -32)	0.80	(0.72 – 0.85)
**Ratios** *(relative to humeral head size)*						
G – Y	-0.45 ± 0.05	-0.46	(-0.58 – -0.35)	(-0.48 – -0.42)	0.80	(0.72 – 0.86)
G – BC	-0.04 ± 0.11	-0.04	(-0.34 – 0.19)	(-0.10 – 0.04)	0.86	(0.81 – 0.90)
G – CC	-0.85 ± 0.13	-0.84	(-1.35 – -0.62)	(-0.93 – -0.76)	0.74	(-0.65 – 0.81)

ICC can be interpreted as follows: poor, < 0.40; fair, 0.41–0.59; good, 0.60–0.74; excellent, 0.75–1.00. Positive values indicated that points were lateral to the glenoid, whereas negative values indicated that points were medial to the glenoid

*Abbreviations*: *ICC* Intraclass Correlation Coefficient

While most measurements differed significantly among men and women, only the ratio of distance for point Y differed significantly among sexes (Table 2).

**Table 2:** Radiographic measurements regrouped by sex

	Men (*N*=50)				Women (*N*=36)				
Variable	Mean ±SD	Median	(range)	(IQR)	Mean ±SD	Median	(range)	(IQR)	*p* value
**Measurements**									
Humeral head size *(mm)*	45.9 ± 2.9	46.3	(36 – 52)	(44 – 48)	40.0 ± 2.5	39.7	(36 – 48)	(38 – 41)	***<.001***
G – Y	-20.2 ± 2.4	-20.1	(-26 – -16)	(-21 – -18)	-18.7 ± 1.9	-18.8	(-24 – -15)	(-20 – -17)	***0.003***
G – BC	-1.0 ± 4.6	-0.6	(-10 – 9)	(-5 – 2)	-2.2 ± 4.5	-2.1	(-13 – 9)	(-5 – -1)	*0.192*
G – CC	-38.6 ± 6.5	-38.1	(-60 – -29)	(-42 – -35)	-34.4 ± 6.0	33.6	(-58 – -23)	(-36 – -31)	***<.001***
**Ratios** *(relative to humeral head size)*									
G – Y	-0.44 ± 0.05	-0.45	(-0.55 – -0.35)	(-0.47 – -0.40)	-0.47 ± 0.04	-0.47	(-0.58 – -0.37)	(-0.49 – -0.45)	***0.006***
G – BC	-0.02 ± 0.10	-0.01	(-0.24 – 0.18)	(-0.09 – 0.05)	-0.05 ± 0.11	-0.06	(-0.34 – 0.19)	(-0.12 – -0.01)	*0.105*
G – CC	-0.84 ± 0.13	-0.85	(-1.25 – -0.62)	(-0.93 – -0.74)	-0.86 ± 0.13	-0.84	(-1.35 – -0.62)	(-0.92 – -0.81)	*0.500*

Positive values indicated that points were lateral to the glenoid, whereas negative values indicated that points were medial to the glenoid

## Discussion

The most important finding of this study was that, out of the 3 anatomic landmarks assessed for mediolateral referencing in shoulder arthroplasty, the consistency was greatest for point Y, with excellent repeatability. Likewise, the consistency of mediolateral ratios (relative to humeral head size) was greatest for point Y, with excellent repeatability. These findings confirm the hypothesis that the mediolateral distance between the Y-plane and the center of the glenoid surface has the greatest consistency and repeatability, with a predictable ratio relative to humeral head size (0.42–0.48 in 50% of shoulders). The present findings could enable surgeons to prescribe appropriate lateralization of the glenoid component using preoperative templating or 3D planning. Future studies should include shoulders with different pathologies to confirm the appropriateness of measurements in cases with severe glenoid bone loss, during revision surgeries, or when humeral head measurements are not reliable.

With the rising number of shoulder arthroplasties performed worldwide, the incidence of complex and revision cases is increasing [21]. In shoulders with glenoid bone loss (degenerative or revision cases), many of the conventional anatomic landmarks such as the base of the coracoid, glenoid surface or glenoid rim, may no longer be discernible, rendering component positioning challenging [1]. The Y-plane could therefore be a suitable reference for preoperative planning; the present study found that in most shoulders the native center of the glenoid surface would be 18 to 21 mm lateral from the Y-plane. Acknowledging that the glenoid surface is 18 to 21 mm laterally from the Y-plane, surgeons would be able to reposition the joint space, adapt their reconstructions with the help of the other remaining landmarks available. Thus the reconstruction would address not only coronal and axial angle deformity, but also restore the accurate and required lateralisation. Furthermore, in RSA, central glenoid defects can be encountered after the removal of the component [9, 10, 19]. Estimation of the native glenoid surface is important to restore anatomy and biomechanics of total shoulder arthroplasty (TSA), and to optimize stability, range of motion and survival of RSA [4, 5, 12, 15, 16].

The Y-plane, corresponding to the most lateral parasagittal image on which the scapular spine is in contact with the scapular body, has already been described for the assessment of fatty infiltration and rotator cuff muscle integrity [8, 18]. The Y-plane is considerably more medial than the BC, and therefore could be less affected in cases with severe glenoid bone loss. There is no standard method to determine the optimal size of metallic or bony augmentation grafts corresponding to the glenoid defect on the CT scan [3, 7], but given that the native center of the glenoid surface would be 18 to 21 mm lateral to the Y-plane in most shoulders (Table 1), the Y-plane could be a suitable reference for preoperative planning; however, the ease and accuracy of identifying the Y-plane in cases of glenoid bone loss or revision surgery remains to be confirmed. Moreover, the ratio of mediolateral distance between points G and Y relative to humeral head size is consistent, suggesting that the appropriate positioning of the glenoid component could also be determined from humeral head size.

The present study has a number of limitations that should be considered when interpreting its findings. First, the sample studied does not represent the population of patients that would require shoulder arthroplasty, though the inclusion and exclusion criteria were deliberately restricted to represent shoulders with little or no scapular deformities, in order to understand how the reference landmarks vary in shoulders with normal bony anatomy. Second, both observers who performed measurements were senior shoulder surgeons, that may not reflect the appreciation and repeatability of junior surgeons or radiologists. Third, because the authors studied pre-existing CT scans acquired to diagnose non-degenerative shoulder pathologies, some of the images did not reach the inferior pole of the scapula, so it was not possible to correct CT orientation in all 3 dimensions prior to selecting points of interest. The authors believe, however, that the 2-dimensional corrections made enable sufficiently accurate identification of the scapular axis, along which most of the measurements were made. Fourth, the measurements and ratios are merely theoretical values, and their utility for preoperative planning and/or intraoperative adjustment of implant positioning remains to be confirmed. The authors plan to perform future studies on shoulders with different pathologies and include measurements from junior surgeons and radiologists to confirm the appropriateness of the measurements and ratios across a wider range of patients and users/clinicians. The authors deemed it important, nevertheless, to report the utility and reliability of the Y-plane on healthy shoulders with no abnormalities to establish normal values, and with measurements from 2 experienced shoulder specialists to eliminate potential sources of errors at the initial step.

## Conclusion

The Y-plane is a reliable CT-based reference for glenoid component positioning in shoulder arthroplasty, with a consistent distance from the center of the glenoid surface, relative to the humeral head size. The Y-plane could therefore be a suitable reference for preoperative planning, given that in most shoulders the native center of the glenoid surface would be 18 to 21 mm lateral from the Y-plane.

## Data Availability

The datasets used and/or analysed during the current study are available from the corresponding author on reasonable request.

## Abbreviations

TSA: Total Shoulder Arthroplasty
RSA: Reverse Shoulder Arthroplasty
CT: Computed Tomography
IRB: Institutional Review Board
DICOM: Digital Imaging and Communication in Medicine
SA: Scapular Axis
ICC: Intra-class & Inter-observer Correlation Coefficients
SD: Standard Deviations
IQR: InterQuartile Ranges
